# Copper Amalgam, Some of Its Mechanical Uses

**Published:** 1892-08

**Authors:** John G. Harper

**Affiliations:** ST. LOUIS, MO.


ARTICLE IV.

### COPPER AMALGAM, SOME OF ITS MECHANICAL USES.

BY JOHN G. HARPER, D.D.S., ST. LOUIS, MO.

**Read before the Missouri State Dental Association, July, 1892.**

The mechanical uses of copper amalgam are quite numerous, for instance cold soldering, dies, etc. First we will consider the making of the amalgam by a simple process. One is as follows:

"Dissolve 10 ounces of sulphate of copper in 2 pints of water, and then precipitate the copper by the introduction of strips of zinc. Wash the precipitate in hot water two or three times, drain off, and for every ounce of precipitated copper add 2 ounces of mercury, add also a little sulphuric acid (say 15 to 20 drops) to aid in the amalgamation of the metals. The finely divided copper and mercury form a paste which sets and becomes intensely hard in the course of a few hours. It should be made up while soft, into little pellets and put away."

Here is another process; you can take your choice:

"To 1 oz. (troy) of pulverized sulphate of copper add 8 fluid ozs. of warm water. This dissolves the salt promptly. A piece of thick bar iron previously brightened by being placed in a pickle bath, should now be immersed in the solution. The formation of solution of sulphate of iron and the precipitation of copper in fine pulp commences immediately. This process continues for several hours, and the copper pulp should occasionally be scraped off the iron plate into the solution with a piece of stick; when no more copper precipitates, the iron plate should be taken out and cleaned for future use.

"The supernatent solution is of a yellowish green color, and when it is reasonably clear by the setting of the precipitate, it should be carefully poured off; sufficient pickle is now poured upon the precipitate, and this is frequently stirred with a glass rod. This is for the purpose of cleaning and purifying the copper pulp, and should be done at intervals for several hours, until no bubbles rise from the agitation. When sufficiently pickled, the acid water is poured off, and the soft pulpy residum is put into a glass or wedgewood mortar, to which is added 1 oz. of mercury, the whole is then rubbed up with a pestle, and prompt and complete amalgamation ensues. After the amalgam is made, it is washed repeatedly. The mass is now squeezed gently through a chamois skin, and the half-squeezed mass is thoroughly triturated in the mortar; it is then squeezed hard, and again tritured, divided into small portions, and is either packed into molds or squeezed into wafers, and allowed to harden. This takes from 12 to 24 hours, and the amalgam is then ready for use, and in the form in which we obtain it from the depots."

The first process is from the *National Druggist*, and the second one from the *Western Dental Journal*. We are also indebted to the *National Druggist* for the following methods of cold soldering:

"The flux consists of 1 part of sodium to 50 parts of mercury. This must be carefully protected from the atmosphere in a glass-stoppered bottle. This has the property of amalgamating any metal with which it comes in contact, forming an adhesive amalgam even on cast iron. First amalgamate the surface to be joined by rubbing them lightly with the flux. This is the equivalent of tinning in the ordinary soldering methods. Then take one (or more as the case may be) of the pellets of copper-amalgam and warm till the mercury begins to exude at the surface, wipe off the exuded drops with a clean rag, and drop the pellets in a small mortar and rub till smooth, or about the consistency of prepared white lead; smear this over one of the surfaces to be joined, and apply the other surface to the latter as quickly as possible. The joint sets so firmly in the course of two and a half or three hours, that only a hammer and cold-chisel (or a degree of heat sufficient to melt ordinary solder) can separate the surfaces."

I called on the editor of the *National Druggist*, to get some further information on the above subject. He told me he had cold-soldered a band-saw, and to his certain knowledge it had lasted for over a year.

Mr. Windhorst, a dental student, used the cold soldering process to replace a tooth on a bridge with satisfaction, also soldered the shank of a mouth mirror, which had been broken, and could not be hot-soldered without taking the glass out.

"For an even stronger and much quicker setting solder, where expense is no item, take the following to replace the copper and mercury (using the same flux):

| | | |
|---|---|---|
| Silver | - - - | 8 parts. |
| Tin | - - - | 10 parts. |
| Bismuth | - - - | 1 part. |
| Platinum | - - | 1 part. |

"Melt together and cast an ingot. Rasp to filings, or otherwise reduce to small particles. When required for use, mix about 3 parts of filings and 1 of mercury in a small martar till it becomes smooth paste. This sets in about fifteen minutes, and cannot be made workable again by heat; it must be mixed as required.

"The omission of the platinum reduces the strength of the solder, and lengthens the time required to harden to about one hour. The omission of bismuth makes a more granular mass, which is better for filling up crevices, with bismuth it is as smooth and plastic as potter's clay. Joints made by this solder are almost inseparable. It is very valuable in repairing surgical and philosophical instruments, the brazing of delicate springs, and in all cases where the application of heat would be hurtful or destructive."

Cold welding has not been a success in my hands. I purchased the sodium amalgam already prepared, shall make some myself according to the formula given, and try that.

The first person to recommend copper-amalgam for dies for crowns, was Dr. J. W. Whipple. He made a depression in a block of lead, filled the depression with softened copper-amalgam, imbedded the formed crown in the soft amalgam, this when hard gave a female die of the cusps.

Dr. Gilmor for some time has been using copper-amalgam dies for forming gold inlays. The cavity of the tooth is properly formed, all undercuts filled, also a portion of the cavity with cement, this when hard is trimmed smooth, the remainder filled with gutta-percha, and a cross cut is made in the surface to act as a guide. An impression is taken of the tooth, the gutta-percha filling removed and properly placed in the impression, being held in place with a little varnish. The impression is filled with copper-amalgam, the rest of the impression is filled with plaster of Paris, and articulation made if it is desired. When the copper-amalgam has hardened, the impression is removed leaving a model of the tooth with the cavity.

Number 120 gold is pressed into the cavity, removed, imbedded in a mixture of plaster of Paris and pumice stone. This gold matrix is filled with small pieces of 20k plate, melted carefully with the blow-pipe, when it is ready for polishing and cementing into the cavity,

Models of teeth for forming clasps made of copper-amalgam are superior to plaster ones.

Models of roots for crowning are more satisfactory if made of copper-amalgam.

Amalgam models of teeth for forming caps for the retention of the teeth or regulating appliances, can be used with a high grade of gold.

These suggestions are given in the hope that they will be a benefit to the profession, and bring these ideas to a number who have heretofore overlooked them when presented in a different form.

The *Dominion Dental Journal*, July, 1892, contains the following hints regarding the mechanical uses of copper-amalgam. They are appended to the original paper in order to make it nearer complete. "Copper-amalgam is very useful for many things in dentistry, besides filling teeth. It may be used for fastening a tooth on a rubber plate, making a full crown for back molars, making matrices for striking up a gold cusp or articulating surface for a gold crown, strengthening or reinforcing plaster models, cusps or any part of the gums or root of a tooth, or a whole tooth may readily be built up in the impression before running the plaster into it. This is often very useful when it is necessary to fit a gold or platinum band round a tooth or root, as the amalgam tooth is quite hard enough to burnish on, while the plaster tooth is frail and would be broken. The amalgam can be saved and used over and over again."--*Western Dental Journal.*

